# A CITIZEN SCIENCE PROJECT ENGAGING NIGERIAN OLDER PERSONS IN A NEIGHBORHOOD ASSESSMENT FOR PHYSICAL ACTIVITY

**DOI:** 10.1093/geroni/igae098.1714

**Published:** 2024-12-31

**Authors:** Michelle Porter, Emmanuel Odeyemi, Stephanie Chesser, Abby King

**Affiliations:** University of Manitoba, Winnipeg, Manitoba, Canada; University of Manitoba, Winnipeg, Manitoba, Canada; University of Manitoba, Winnipeg, Manitoba, Canada; Stanford University, Stanford, California, United States

## Abstract

A main tenet of age-friendly principles is that the voices of older people should be guiding improvements to their communities. The purpose of this collaborative citizen science project was to examine the age-friendliness of a neighborhood in Lagos Nigeria in terms of its characteristics to enable physical activity. Citizen scientists (13 older adults, seven men and six women, 65 to 86 years old) were involved in data collection, analysis, and priority setting. For data collection, they used the Stanford Healthy Neighborhood Discovery Tool application on a tablet to record 156 photos and 151 commentaries (text and audio) of neighborhood barriers and facilitators to physical activity. The collaborative analysis and priority setting process led to the following facilitators being identified: pedestrian and traffic facilities (e.g., traffic lights, walkways); green areas and parks; multi-generational community features (e.g., programs / facilities); opportunities for social connection (e.g., neighborhood associations, churches); safety of destinations and services; and public toilets. Barriers included: hazardous walkways / traffic; noise pollution; refuse; selling of public parks; crime (e.g., kidnapping, criminal hideouts); no safe drinking water; and ageism. The identified priorities for change in this Nigerian neighborhood included both social and physical aspects of the environment: social connectivity; improved pedestrian and traffic facilities; and green and beautiful environments. By meaningfully engaging older adults, this type of approach shows promise for communities in making age-friendly changes.

